# A Hybrid Laparoscopic–Endoscopic Approach Enabling Endoscopic Submucosal Dissection for Early Gastric Cancer in a Patient with a Large Hiatal Hernia: A Case Report

**DOI:** 10.70352/scrj.cr.26-0372

**Published:** 2026-07-29

**Authors:** Byonggu An, Hiromitsu Ban, Yasumitsu Oe, Toru Imagami, Akira Sogawa, Nobuyuki Takao, Takashi Matsunaga, Atsushi Hirata, Akiyoshi Mizumoto, Shizuki Takemura, Tetsuya Abe, Toru Miyake, Shinnosuke Yamada, Takeshi Togawa

**Affiliations:** 1 Department of Digestive Surgery, Omi Medical Center, Kusatsu, Shiga, Japan; 2 Department of Surgery, Shiga University of Medical Science, Otsu, Shiga, Japan; 3 Department of Gastroenterology, Omi Medical Center, Kusatsu, Shiga, Japan; 4 Department of Diagnostic Pathology, Omi Medical Center, Kusatsu, Shiga, Japan; 5 Department of Gastroenterological Surgery, Aichi Cancer Center Hospital, Nagoya, Aichi, Japan

**Keywords:** early gastric cancer, endoscopic submucosal dissection, hiatal hernia, hybrid approach, laparoscopic surgery

## Abstract

**INTRODUCTION:**

Endoscopic submucosal dissection (ESD) is a standard treatment for early gastric cancer; however, severe anatomical distortion caused by a large hiatal hernia can render the procedure technically infeasible. We report a case in which laparoscopic reduction enabled successful ESD in a 1-stage hybrid procedure.

**CASE PRESENTATION:**

An older woman with a large type III hiatal hernia was diagnosed with early gastric cancer in the antrum, with most of the stomach herniated into the thoracic cavity. ESD was attempted but proved technically infeasible because of poor endoscopic maneuverability due to the intrathoracic displacement of the stomach. A 1-stage hybrid approach was therefore planned for treatment. With the patient under general anesthesia, laparoscopic reduction and posterior crural closure were performed to restore the normal hiatal anatomy. ESD was then performed under more stabilized conditions that allowed en bloc resection, while intraoperative endoscopy simultaneously enabled assessment of luminal narrowing at the esophagogastric junction. The anterior wall of the upper gastric body was fixed to the abdominal wall to prevent recurrence of the hernia. The patient’s postoperative course was uneventful. Histopathological examination of the resected specimen confirmed the curative resection of an intramucosal adenocarcinoma (pT1a, ly0, v0, HM0, VM0). No recurrence was observed during 3 years of follow-up.

**CONCLUSIONS:**

A hybrid laparoscopic–endoscopic approach can enable ESD in otherwise technically infeasible cases by restoring anatomical configuration and stabilizing endoscopic maneuverability. This strategy may provide a safe and minimally invasive 1-stage treatment option.

## Abbreviation

ESD
: endoscopic submucosal dissection

## INTRODUCTION

ESD has been established as a standard, minimally invasive treatment for early gastric cancer, enabling curative resection while preserving gastric function.^1)^ However, successful ESD requires stable endoscopic maneuverability and an appropriate anatomical configuration, and certain conditions may render the procedure technically difficult or infeasible.^2)^

Hiatal hernia, particularly when large and associated with intrathoracic migration of the stomach, can cause marked anatomical distortions.^3)^ These distortions change the orientation of organs and destabilize the placement of the endoscope, resulting in poor visualization and limited control over the instrument. Consequently, even lesions that satisfy the oncological criteria for ESD may become technically infeasible.

The optimal management strategy for patients with early gastric cancer complicated by severe hiatal hernia remains unclear, particularly when ESD cannot be performed under standard conditions. Moreover, reports describing strategies to enable ESD in such anatomically challenging settings remain limited.^3,4)^

We report a case of early gastric cancer associated with a large type III hiatal hernia in which ESD was attempted but proved technically infeasible because of anatomical distortions. A 1-stage hybrid approach, consisting of laparoscopic reduction and hiatal repair followed by ESD, enabled curative treatment under stabilized conditions. This case highlights a potential strategy for facilitating ESD and expanding its applicability to cases with severe anatomical abnormalities.

## CASE PRESENTATION

An older woman in her 80s was referred to our institution (Omi Medical Center) for evaluation of a gastric lesion detected during routine examination. She had a medical history of hypertension, dementia, and a known large type III hiatal hernia. She had previously undergone surgeries for gallbladder cancer (pT1b[MP]N0M0, fStage I) and colorectal cancer (pT2[SS]N0M0, fStage II). She was asymptomatic and able to tolerate oral intake.

**Fig. 1:**
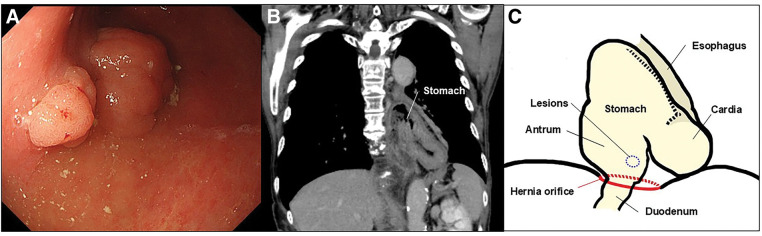
Preoperative findings. (**A**) Upper gastrointestinal endoscopy revealed 2 elevated lesions (type 0–I) on the anterior wall of the antrum. (**B**) Contrast-enhanced CT demonstrated a large type III hiatal hernia, with most of the stomach, including the antrum, herniated into the thoracic cavity. (**C**) Schematic diagram of the type III hiatal hernia. The red circle indicates the hernia orifice, whereas the blue dotted circle indicates the location of the gastric tumor within the herniated stomach.

Upper gastrointestinal endoscopy revealed 2 elevated lesions (type 0–I) located on the anterior wall of the antrum (**Fig. 1A**). A histopathological examination initially revealed group 3 lesions (according to the Japanese classification of gastric carcinoma),^5)^ which were managed with surveillance; however, a repeat biopsy revealed progression to group 4 lesions with further growth of the lesions, a finding increasingly suspicious for early gastric cancer, and indicating the need for ESD.

ESD was attempted; however, it proved technically infeasible because severe anatomical distortion caused by the large hiatal hernia resulted in poor endoscopic maneuverability, unstable visualization of the lesion, and difficulty maintaining adequate gastric insufflation, thereby preventing safe and reliable submucosal dissection. Contrast-enhanced CT demonstrated a large type III hiatal hernia, with most of the stomach, including the antrum, herniated into the thoracic cavity (**Fig. 1B** and **1C**). Based on CT and endoscopic findings, a multidisciplinary team decided to proceed with a 1-stage hybrid approach combining laparoscopic surgery and ESD.

**Fig. 2:**
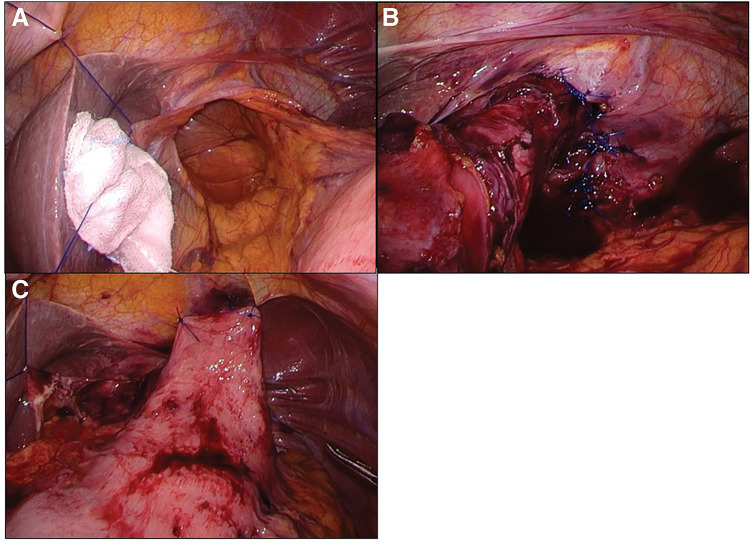
Intraoperative findings. (**A**) The herniated stomach was reduced into the abdominal cavity. (**B**, **C**) Posterior crural closure was performed by suturing the diaphragmatic crura. After ESD, the esophagus was then fixed to both crura, followed by anterior fixation of the upper gastric body to the abdominal wall to prevent recurrence of the hiatal hernia. ESD, endoscopic submucosal dissection

**Fig. 3:**
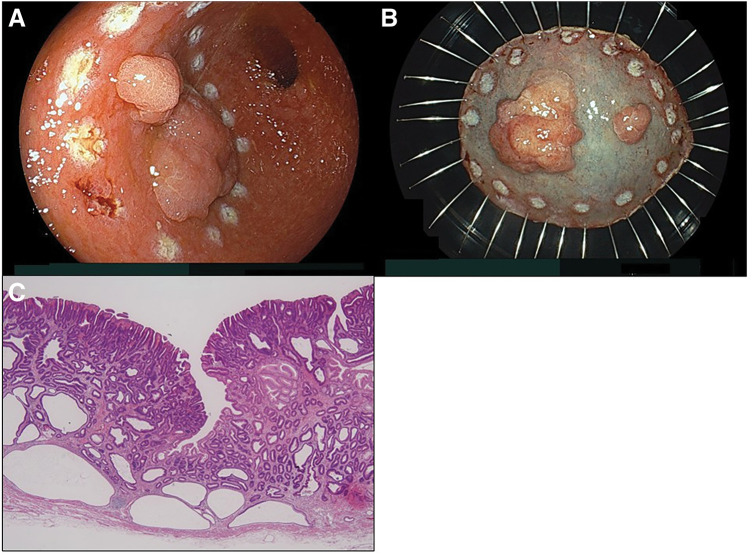
Endoscopic and histopathological findings. (**A**, **B**) ESD was performed under stabilized conditions, allowing en bloc resection of both lesions. (**C**) Histopathological examination revealed a well-differentiated tubular adenocarcinoma confined to the mucosa (pT1a), without lymphovascular invasion (ly0, v0) and negative horizontal and vertical margins (HM0, VM0), consistent with curative resection. ESD, endoscopic submucosal dissection

**Supplementary Figure 1** depicts the overall treatment strategy and procedural flow. With the patient under general anesthesia, laparoscopic surgery was performed. Five trocars were placed in a configuration similar to that used for standard laparoscopic gastric cancer surgery: a 12-mm umbilical trocar; 5-mm trocars in the bilateral subcostal regions along the anterior axillary lines; a 12-mm trocar in the left mid-abdomen along the midclavicular line; and a 5-mm trocar in the right mid-abdomen along the midclavicular line. After placement of the ports, intra-abdominal adhesions related to the previous surgeries were dissected to secure an adequate operative field. The herniated stomach was reduced into the abdominal cavity (**Fig. 2A**), revealing a markedly enlarged esophageal hiatus with an associated hernia sac measuring approximately 7 × 8 cm. The esophagus was then mobilized while preserving the vagal nerves. Posterior crural closure was performed using interrupted 3-0 Prolene sutures (Ethicon, Somerville, NJ, USA), leaving an approximately 5-mm hiatus. Immediately before endoscopic intervention, the jejunum distal to the ligament of Treitz was temporarily clamped with laparoscopic atraumatic forceps to help maintain adequate gastric distension. ESD was subsequently performed under stable conditions, allowing en bloc resection of both lesions (**Fig. 3A** and **3B**). Following completion of the ESD, the esophagus was sutured to both the right and left diaphragmatic crura, and the anterior wall of the upper gastric body was fixed to the abdominal wall (**Fig. 2B** and **2C**). Fundoplication was not performed because the patient did not have preoperative symptoms of gastroesophageal reflux.

The patient’s postoperative course was uneventful, and she resumed oral intake without complications. No intraoperative complications, including perforation or significant bleeding, were observed. Histopathological examination of both resected lesions revealed well-differentiated tubular adenocarcinomas confined to the mucosa (pT1a), without lymphovascular invasion (ly0, v0) and with negative horizontal and vertical margins (HM0, VM0). The findings were consistent with curative resection (**Fig. 3C**). During 3 years of follow-up, the patient remained free of recurrence of both the gastric cancer and hiatal hernia (**Supplementary Fig. 2**).

## DISCUSSION

Our case report highlights 2 major clinical points. First, severe anatomical distortion caused by a large hiatal hernia can render ESD technically infeasible by impairing visualization, endoscopic maneuverability, and adequate luminal distension. Second, a 1-stage hybrid approach involving laparoscopic reduction and hiatal repair prior to ESD can restore normal anatomy and enable safe and effective endoscopic resection.

The coexistence of early gastric cancer with a large hiatal hernia presents a therapeutic challenge. While ESD has become a standard minimally invasive treatment for early gastric cancer,^1)^ successful ESD requires adequate visualization, stable endoscopic maneuverability, and an appropriate anatomical configuration. Previous studies have identified tumor size, location, and patient-related factors as predictors of technical difficulties with the use of ESD.^2)^ However, these factors do not account for anatomical distortion. In cases of large hiatal hernia, particularly type III hernias with intrathoracic migration of the stomach, anatomical distortions disrupt the spatial orientation and destabilize the endoscopic field, resulting in impaired visualization and limited control of the instrument, thereby reducing the feasibility of ESD. In the present case, ESD was attempted but proved technically infeasible because of severe anatomical distortions, which resulted in unstable visualization of the lesion, compromised endoscopic maneuverability, and inadequate luminal distension.

To overcome the limitations of ESD in patients with severe hiatal hernias and gastric cancer, we adopted a 1-stage hybrid approach in which laparoscopic reduction and posterior crural closure were performed prior to ESD. Restoration of the hiatal anatomy improved the stability of the endoscopic field and facilitated precise endoscopic manipulation. Furthermore, crural closure may help maintain adequate gastric distension during endoscopic procedures by stabilizing the anatomical configuration and reducing air leakage from the stomach, thereby improving endoscopic visualization and maneuverability. Performing ESD after crural closure also allowed the intraoperative assessment of luminal narrowing at the esophagogastric junction.

A limited number of reports have described successful ESD in patients with markedly altered gastric anatomy, including upside-down stomachs,^3)^ as well as laparoscopic–endoscopic approaches for gastric tumors associated with hiatal hernia.^4)^ These reports suggest that severe anatomical displacement can adversely affect endoscopic orientation, visualization, and maneuverability. However, the reported approaches primarily focused on overcoming technical difficulties during ESD itself or performing cooperative resections and did not address correction of the underlying anatomical distortion. To our knowledge, few reports have described a 1-stage hybrid laparoscopic–endoscopic strategy in which hiatal hernia repair was performed first to restore normal anatomy and subsequently enable curative ESD. The present case therefore demonstrates the feasibility of a 1-stage hybrid laparoscopic–endoscopic strategy for selected patients in whom anatomical distortion renders conventional ESD technically infeasible.

Our strategy differs from conventional approaches in that surgical intervention was used not as definitive oncologic treatment, but as a means to facilitate endoscopic therapy. Previous reports have described surgical intervention following ESD to manage complications, such as repair of a laparoscopic hiatal hernia with pyloroplasty for post-ESD stenosis.^6)^ Additionally, cooperative laparoscopic and endoscopic surgery has been used as a salvage procedure when ESD cannot be completed.^7)^ In contrast, our approach represents a preemptive strategy aimed at enabling ESD and avoiding treatment failure or more invasive procedures.

The patient had dementia and required deep sedation or general anesthesia for safe endoscopic intervention. Considering her advanced age and the need to minimize invasive procedures, a 1-stage approach during a single session under general anesthesia was considered preferable. Several alternative treatment strategies could also have been considered for our patient. Gastrectomy would have provided definitive treatment for both gastric cancer and the concomitant hiatal hernia; however, it would have represented a substantially more invasive procedure than necessary for an intramucosal lesion amenable to endoscopic resection. A staged approach, consisting of hiatal hernia repair followed by ESD at a later date, was also considered. However, this strategy would have required 2 separate procedures and hospitalizations, potentially increasing the overall treatment burden. Observation was not considered appropriate because the lesion had demonstrated interval growth during surveillance and was highly suspicious for early gastric cancer. In addition, the patient’s family strongly preferred definitive treatment rather than continued observation. Therefore, active treatment was considered warranted. The 1-stage hybrid laparoscopic–endoscopic approach, in contrast, allowed the simultaneous correction of the anatomical distortion caused by the hiatal hernia and a curative endoscopic resection of the tumor, while preserving the stomach. Accordingly, we decided that this strategy would, in this case, provide the optimal balance between curative oncological curability and procedural invasiveness.

From a technical standpoint, the sequence of procedures is critical. For our patient, ESD was performed after reduction and crural closure, which not only stabilized endoscopic maneuverability but also enabled the intraoperative assessment of potential stenosis following hiatal repair. Finally, the esophagus was fixed to the diaphragmatic crura to maintain its anatomical position, and the anterior wall of the upper gastric body was fixed to the abdominal wall as a gastropexy to prevent re-migration of the stomach into the thoracic cavity.^8)^

The applicability of this approach may vary according to tumor location. In our case, the lesion was located in the antrum, and restoration of normal gastric anatomy after reduction of the hiatal hernia provided a stable endoscopic field that facilitated safe ESD. In contrast, lesions located near the esophagogastric junction or cardia may present additional technical challenges because of their proximity to the hiatal repair site and the potential risk of luminal narrowing after crural closure. Furthermore, oncological and functional considerations may differ for lesions involving the esophagogastric junction. These factors should be taken into account when considering this strategy. Careful patient selection is essential, particularly in cases with a large hiatal hernia causing significant intrathoracic migration of the stomach. Our approach may expand the indications for ESD in selected patients with severe anatomical distortions.

## CONCLUSIONS

In selected cases, laparoscopic reduction and hiatal repair may enable ESD in otherwise technically infeasible situations by restoring normal anatomy and improving endoscopic maneuverability. A 1-stage hybrid laparoscopic–endoscopic approach may represent a safe and minimally invasive treatment option for early gastric cancer associated with severe hiatal hernia.

## SUPPLEMENTARY MATERIALS

Supplementary Fig. 1 Flowchart of the hybrid laparoscopic–endoscopic procedure.
Laparoscopic reduction of the herniated stomach and posterior crural closure were performed to restore the hiatal anatomy and stabilize endoscopic maneuverability, which enabled subsequent ESD. Intraoperative assessment of luminal patency was performed after hiatal repair, followed by fixation of the esophagus to the diaphragmatic crura and anterior fixation of the upper gastric body to prevent recurrence of the hiatal hernia.

Supplementary Fig. 2 Postoperative CT findings at 3-year follow-up. Contrast-enhanced CT (coronal view) demonstrated that the stomach remained in the intra-abdominal position without recurrence of the hiatal hernia.
