# Supplementary figures and images for: A Hybrid Laparoscopic–Endoscopic Approach Enabling Endoscopic Submucosal Dissection for Early Gastric Cancer in a Patient with a Large Hiatal Hernia: A Case Report
Source: Surg Case Rep. 2026 Jul 29;12(1):26-0372. doi: 10.70352/scrj.cr.26-0372 (PMC13425071; doi:10.70352/scrj.cr.26-0372)

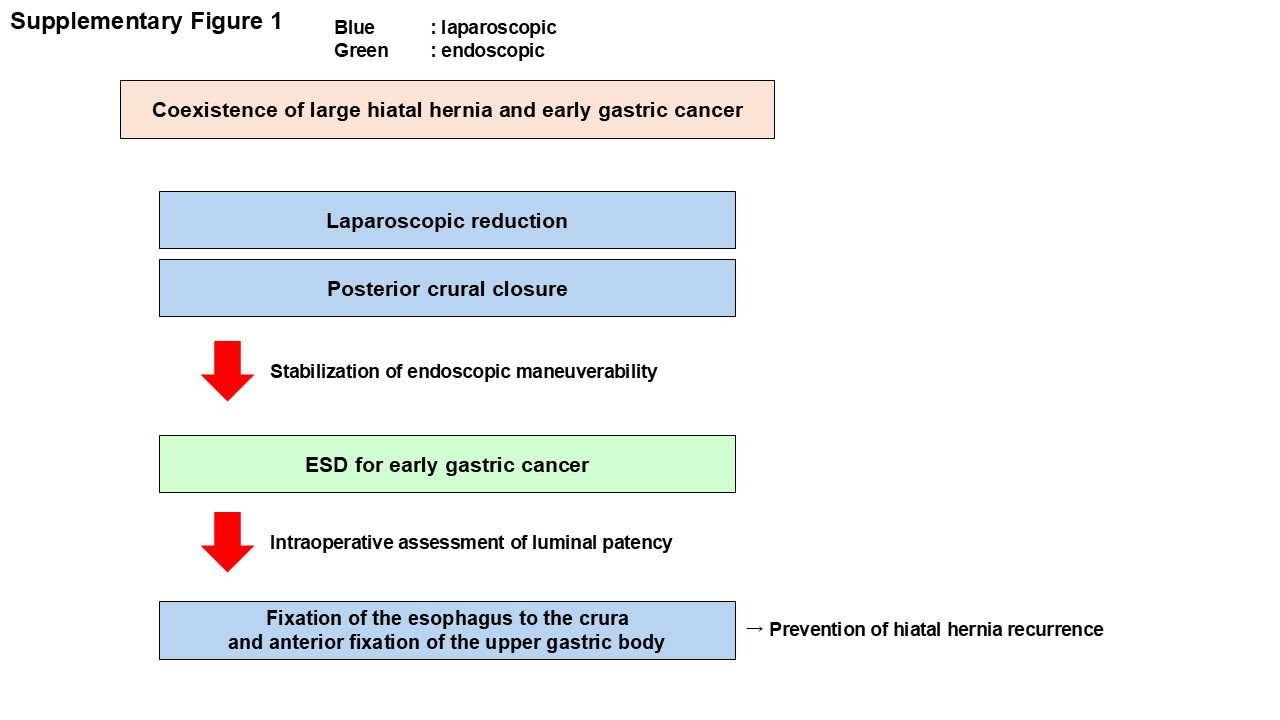

Supplement: Supplementary Fig. 1 — Flowchart of the hybrid laparoscopic–endoscopic procedure. Laparoscopic reduction of the herniated stomach and posterior crural closure were performed to restore the hiatal anatomy and stabilize endoscopic maneuverability, which enabled subsequent ESD. Intraoperative assessment of luminal patency was performed after hiatal repair, followed by fixation of the esophagus to the diaphragmatic crura and anterior fixation of the upper gastric body to prevent recurrence of the hiatal hernia. [file scr-12-01-26-0372-s001.jpg]

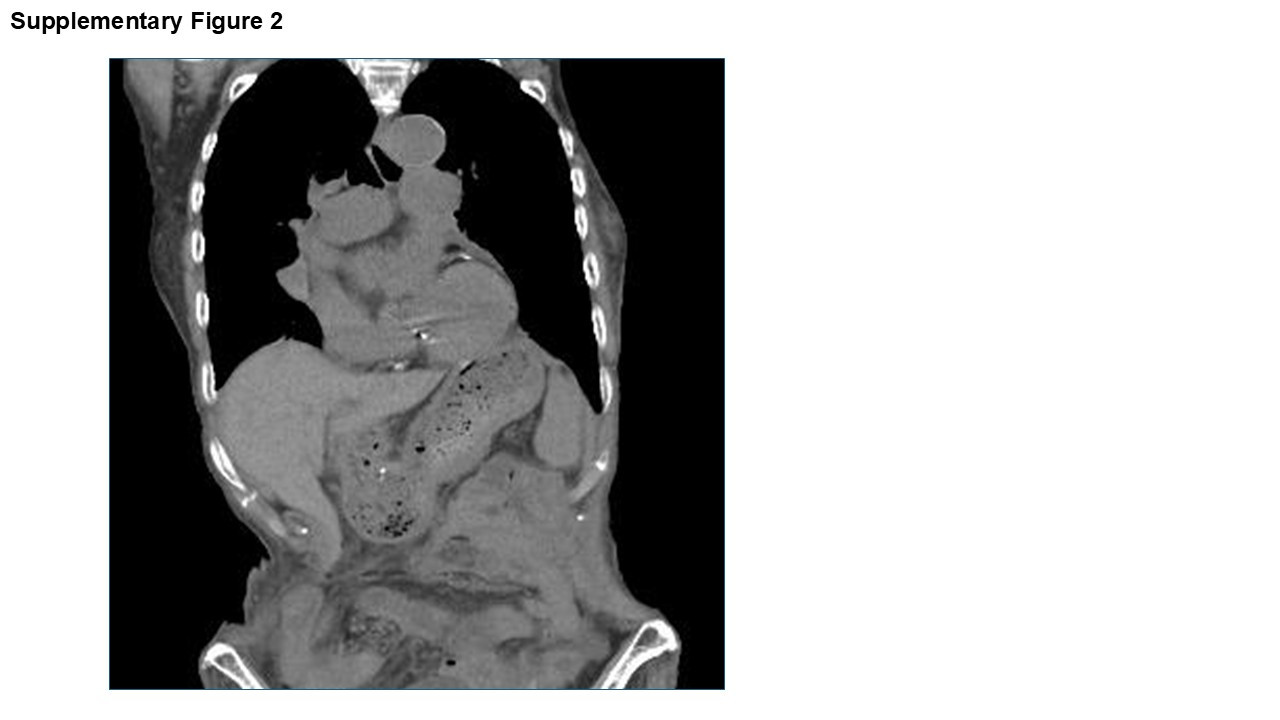

Supplement: Supplementary Fig. 2 — Postoperative CT findings at 3-year follow-up. Contrast-enhanced CT (coronal view) demonstrated that the stomach remained in the intra-abdominal position without recurrence of the hiatal hernia. [file scr-12-01-26-0372-s002.jpg]
